# Supplementary material for: Synthesis, Photophysics, and Solvatochromic Studies of an Aggregated-Induced-Emission Luminogen Useful in Bioimaging
Source: Sensors (Basel). 2019 Nov 12;19(22):4932. doi: 10.3390/s19224932 (PMC6891498; doi:10.3390/s19224932)
Supplement: Supplementary file 1 [file sensors-19-04932-s001.pdf]

sensors

Supporting Materials

Synthesis, Photophysics, and Solvatochromic Studies of an Aggregated-Induced-Emission Luminogen Useful in Bioimaging

Laura Espinar-Barranco [1], Marta Meazza [2], Azahara Linares-Perez [3], Ramon Rios [2], Jose Manuel Paredes [1,*] and Luis Crovetto [1,*]

[1] Department of Physical Chemistry, University of Granada, Unidad de Excelencia en Quimica Aplicada a Biomedicina y Medioambiente (UEQ), Cartuja Campus, 18071 Granada, Spain; lauraebe@correo.ugr.es
[2] School of Chemistry, University of Southampton, Highfield Campus, Southampton SO17 1BJ, UK; M.Meazza@soton.ac.uk (M.M.); R.Rios-Torres@southampton.ac.uk (R.R.)
[3] Department of Biochemistry and Molecular Biology II, Faculty of Pharmacy, University of Granada, Cartuja Campus, 18071 Granada, Spain; azaharalinares@ugr.es
* Correspondence: jmparedes@ugr.es (J.M.P.); luiscrovetto@ugr.es (L.C.)

Received: 10 October 2019; Accepted: 7 November 2019; Published: 12 November 2019

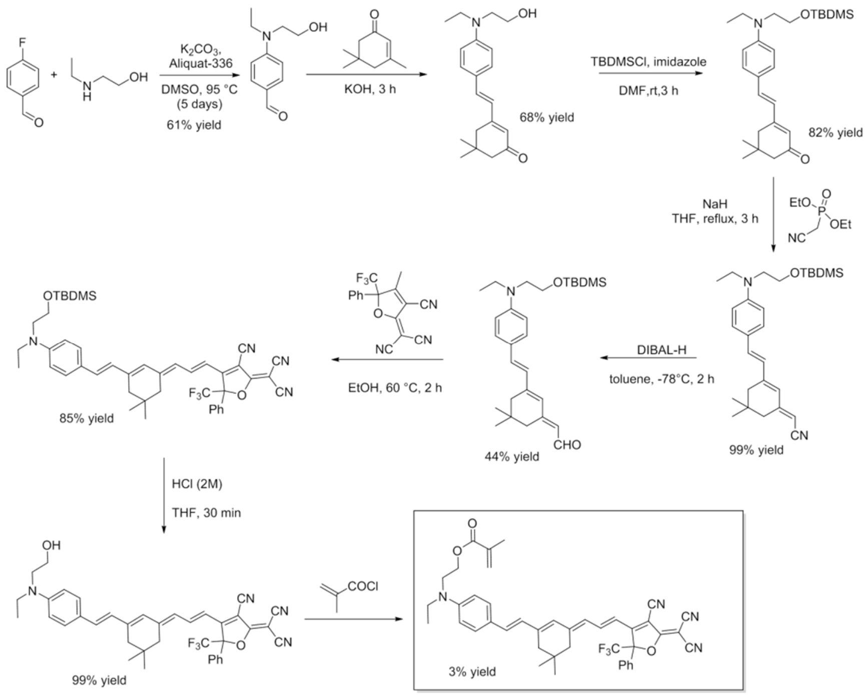

Scheme S1. Synthesis of dye: 2-((4-((*E*)-2-((*E*)-3-((*E*)-3-(4-cyano-5-(dicyanomethylene)-2-phenyl-2-(trifluoromethyl)-2,5-dihydrofuran-3-yl)allylidene)-5,5-dimethylcyclohex-1-en-1-yl)vinyl)phenyl)(ethyl)amino)ethyl methacrylate.

Quantum Yield calculation

The relative fluorescence quantum yield values were determined using the following formula [1]:

$$\Phi = \Phi_R \cdot \frac{I}{I_R} \cdot \frac{OD_R}{OD} \cdot \frac{n^2}{n_R^2}$$

where Φ and Φ_R denote the fluorescence quantum yield of the sample and the reference, respectively, I and I_R the integrated fluorescence spectra of the sample and the reference, OD and OD_R the absorption at the excitation wavelength of the sample and the reference and n and n_R the refractive index of the solvent where the sample and reference are dissolved. As references, we have used Nile Blue A in EtOH (Φ = 0.27) [2]. The samples were excited at the maximum absorption of each solvent.

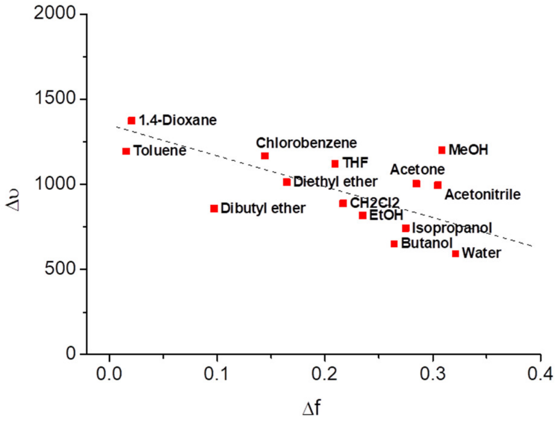

Figure S1. Lippert–Mataga representation of orientation polarizability of PEMC dissolved in different solvents.

Lippert–Mataga equation

$$\overline{\upsilon_A} - \overline{\upsilon_F} = \frac{2}{hc}\left(\frac{\varepsilon - 1}{2\varepsilon + 1} - \frac{n^2 - 1}{2n^2 + 1}\right)\frac{(\mu_E - \mu_G)^2}{a^3} + k$$

In the Lippert–Mataga equation, h is the Planck constant, c represents the light speed in vacuum, a is the radius of the cavity where the dye is allocated, $\overline{\upsilon_A}$ and $\overline{\upsilon_F}$ are the absorption and emission wavenumber, respectively, and k is a constant representing the difference between the absorption and emission wavenumbers in the vacuum.

$$\Delta f = \frac{\varepsilon - 1}{2\varepsilon + 1} - \frac{n^2 - 1}{2n^2 + 1}$$

Orientation polarizability (Δf) is the combination of both parameters as indicated in the equation and is included in the Lippert–Mataga equation.

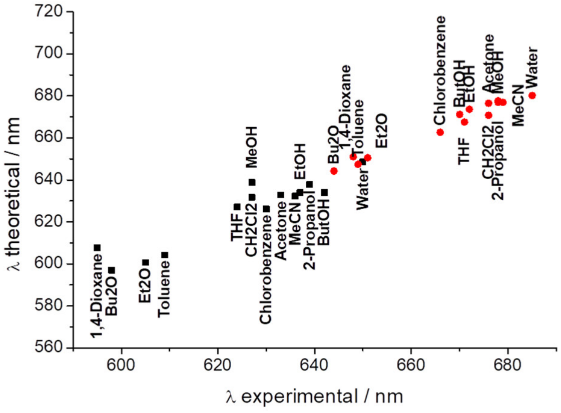

Figure S2. Experimental and theoretical wavelength (obtained from the Catalan approach data) of the 14 solvents used.

Catalan equation

$$A = A_0 + b\,SA + c\,SB + d\,SP + e\,SdP$$

The analysis is based on four empirical solvent scales: polarizability (*SP*), dipolarity (*SdP*), acidity (*SA*), and basicity (*SB*), hence taking into account both general effects and specific hydrogen bonding features of the solvents.

Where A is a solvent-dependent physicochemical property in a specific solvent, A_0 is the statistical quantity corresponding to the value of the property in the gas phase and b to e are the regression coefficients describing the sensitivity of property A to the different solute-solvent interactions.

Table S1. Estimated coefficients ± standard errors and correlation coefficient (r) for the multilinear regression analyses of $\tilde{\nu}_{abs}$ and $\tilde{\nu}_{em}$ The estimates are expressed in cm^{-1}.

	A_0	b (SA)	c (SB)	d (SP)	e (SdP)	r
$\tilde{\nu}_{abs}$	19501 ± 970	-401 ±<br>180	-606 ±<br>271	-3125 ±<br>1157	-1518<br>±240	0.94<br>20
	18803 ± 1071		-357 ±<br>288	-2213 ±<br>1262	-1724 ±<br>258	0.91<br>19
	17819 ±717	-235 ±<br>191		-1266 ±<br>941	-1367 ±<br>269	0.91<br>16
	16919 ± 207	-229 ±<br>211	-81 ±237		-1274 ±<br>278	0.89<br>74
	16484 ± 1804	-840 ±<br>354	-123 ±<br>555	-365 ±<br>2288		0.66<br>02
	16945 ± 153				-1451 ±<br>211	0.88<br>56
	15013 ± 1312			1384 ±<br>1898		0.19<br>83
	15840 ± 229		297 ± 460			0.17<br>62
	16169 ±112	-793 ±				0.65

		251				83
$\tilde{\nu}_{em}$	16658 ± 432	-89 ± 80	-325 ± 121	-1093 ± 515	-1112 ± 107	0.9736
	16504 ± 413		-269 ± 111	-891 ± 487	-1158 ± 99	0.9703
	15758 ± 342	0 ± 91		-98 ± 449	-1031 ± 128	0.9540
	15755 ± 84	-29 ± 86	-141 ± 96		-1027 ± 113	0.9615
	14448 ± 1236	-410 ± 243	30 ± 380	930 ± 1567		0.6175
	15685 ± 65				-1022 ± 89	0.9538
	13901 ± 821			1590 ± 1188		0.3480
	14955 ± 152		95 ± 305			0.0858
	15114 ± 79	-466 ± 176				0.5915

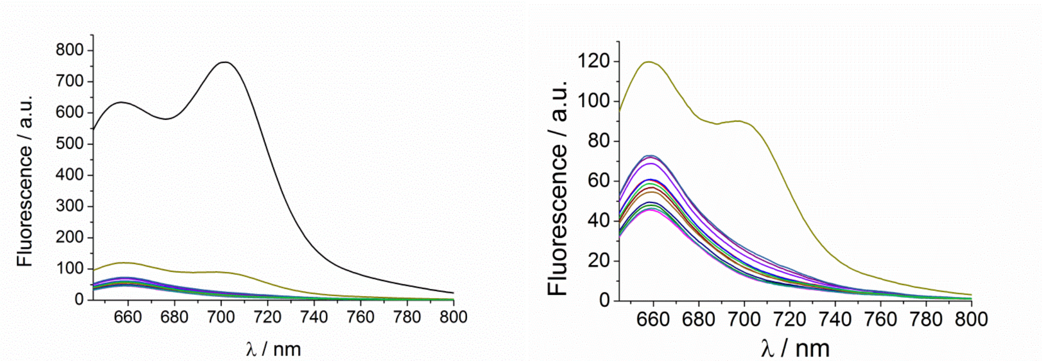

Figure S3. Fluorescence spectra in ethanol as solvent at different PEMC concentrations. (Top) Including 5 x 10^{-3} M (highest value) to see the AIE effect. (Bottom) Without 5x10^{-3} M to see in detail the Kavanagh law.

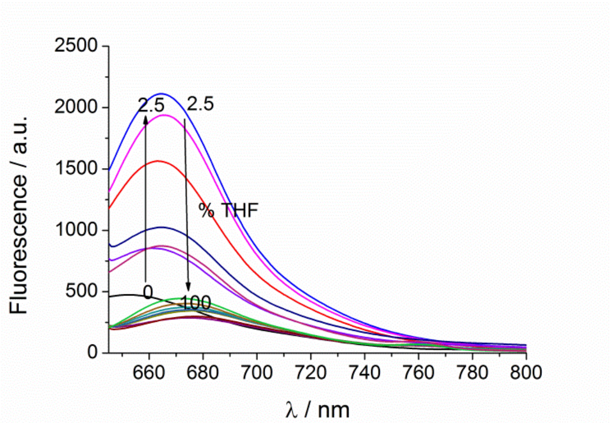

Figure S4. Fluorescence spectrum of PEMC in the presence of increasing proportions of THF to water.

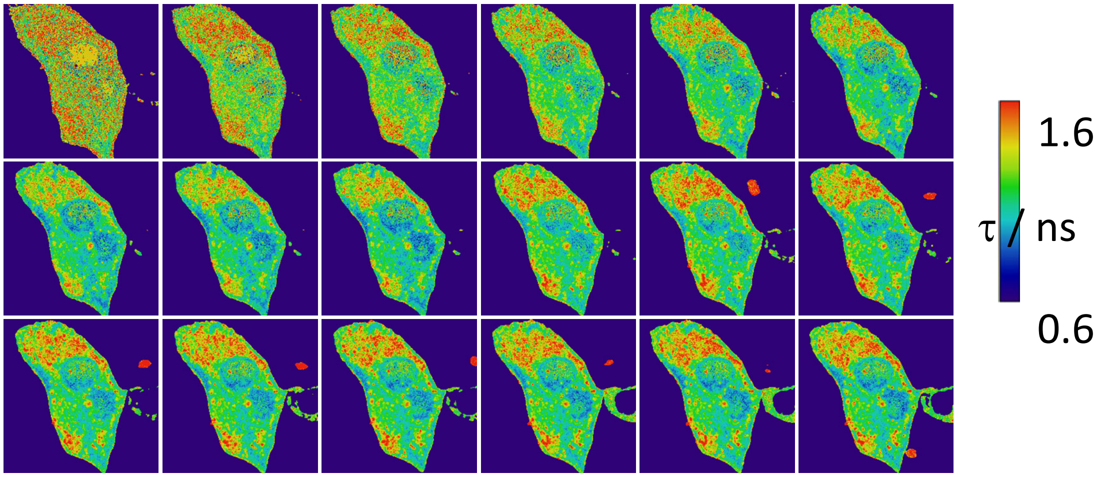

Figure S5. FLIM images of input kinetics of PEMC in MDA-MB-231 cells.

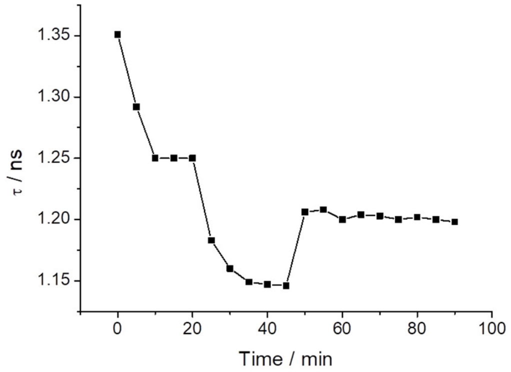

Figure S6. Fluorescence lifetimes recovered from the input kinetics of PEMC in MDA-MB-231 cells from Figure S3.

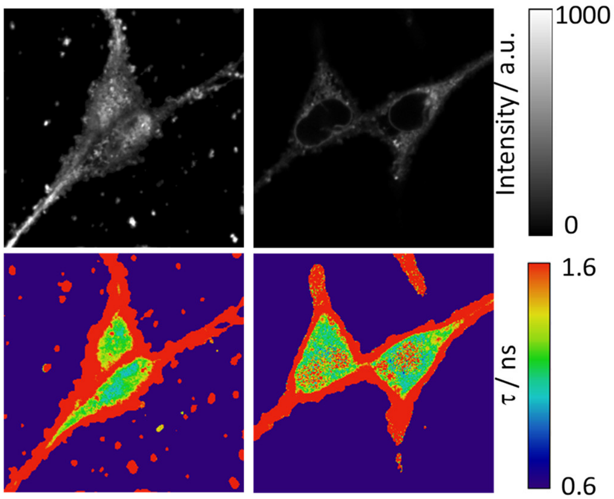

Figure S7. FLIM images of PEMC in HEK cells.

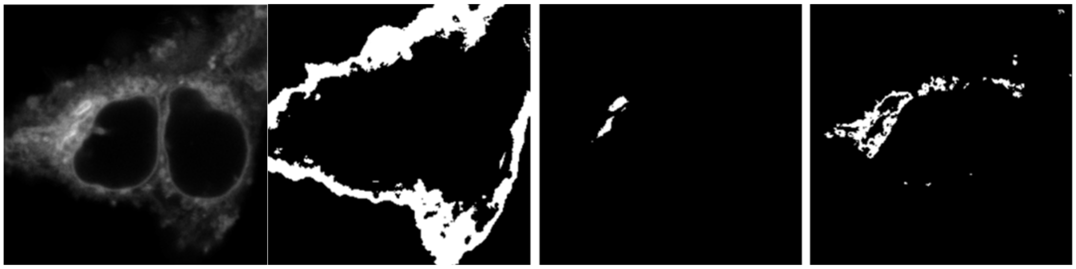

Figure S8. Intensity images of different organelles isolated by intensity threshold.

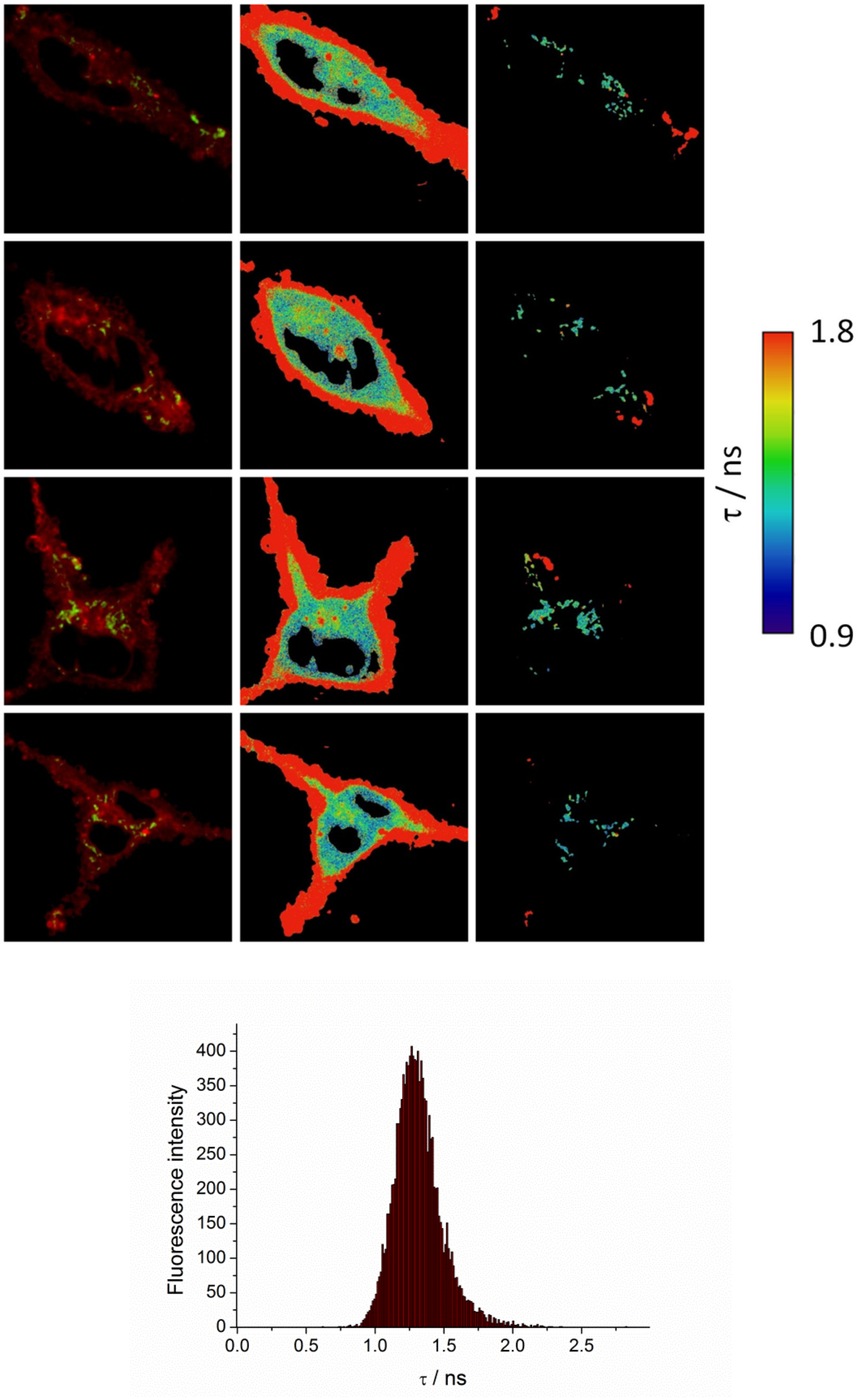

Figure S9. (**Up**) Fluorescence intensity images maps of HEK cells with MitoTracker Green and PEMC (**left**), fluorescence lifetime of PEMC separating the red signal (**in the middle**) and isolating the colocation region (**right**). (**Bottom**) Histograms of PEMC in the mitochondria region of the cells.

Reference

1. Lakowicz, J.R. *Principles of Fluorescence Spectroscopy*; 3rd ed.; Springer: New York, NY, USA, 2006.

2. Brouwer, A.M. Standards for photoluminescence quantum yield measurements in solution (IUPAC Technical Report). *Pure Appl. Chem.* **2011**, *83*, 2213–2228, doi:10.1351/pac-rep-10-09-31.
